# Design and Development of Metasurface Materials for Enhancing Photodetector Properties

**DOI:** 10.1002/advs.202402530

**Published:** 2024-07-05

**Authors:** Renquan Guan, Hao Xu, Zheng Lou, Zhao Zhao, Lili Wang

**Affiliations:** ^1^ State Key Laboratory for Superlattices and Microstructures Institution of Semiconductors Chinese Academy of Sciences Beijing 100083 China; ^2^ Center of Materials Science and Optoelectronic Engineering University of Chinese Academy of Sciences Beijing 100049 China; ^3^ Faculty of Physics Northeast Normal University Changchun 130024 China

**Keywords:** artificial material, electromagnetic wave, metaphotodetectors, metasurface, special properties

## Abstract

Recently, metasurface‐based photodetectors (metaphotodetectors) have been developed and applied in various fields. Metasurfaces are artificial materials with unique properties that have emerged over the past decade, and photodetectors are powerful tools used to quantify incident electromagnetic wave information by measuring changes in the conductivity of irradiated materials. Through an efficient microstructural design, metasurfaces can effectively regulate numerous characteristics of electromagnetic waves and have demonstrated unique advantages in various fields, including holographic projection, stealth, biological image enhancement, biological sensing, and energy absorption applications. Photodetectors play a crucial role in military and civilian applications; therefore, efficient photodetectors are essential for optical communications, imaging technology, and spectral analysis. Metaphotodetectors have considerably improved sensitivity and noise‐equivalent power and miniaturization over conventional photodetectors. This review summarizes the advantages of metaphotodetectors based on five aspects. Furthermore, the applications of metaphotodetectors in various fields including military and civil applications, are systematically discussed. It highlights the potential future applications and developmental trends of metasurfaces in metaphotodetectors, provides systematic guidance for their development, and establishes metasurfaces as a promising technology.

## Graphical abstract

Metasurface‐based photodetectors play a crucial role in various fields. Applying metasurfaces with unique properties to photodetectors can significantly enhance their properties, leading to the miniaturization of traditional photodetectors. This advancement is particularly important for applications in optical communication, imaging technology, and spectral analysis. Therefore, metasurfaces hold great promise for the development of advanced photodetectors.

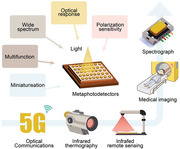

## 1 Introduction

Metamaterials are artificial materials that possess unique physical properties, achieved through the microstructural design of materials.^[^ ^1^, ^2^, ^3^, ^4^, ^5^ ^]^ When the thickness of the metamaterial is smaller than the wavelength of the manipulated electromagnetic waves, it is called a metasurface.^[^ ^6^, ^7^, ^8^ ^]^ Such metasurfaces offer flexible and efficient control over various electromagnetic wave characteristics, including polarization, amplitude, phase, propagation mode, and polarization mode.^[^ ^9^, ^10^ ^]^ Metasurfaces have demonstrated distinctive advantages in various applications, such as holographic projection, stealth, biological image enhancement, biosensing, and energy absorption.^[^ ^11^, ^12^, ^13^, ^14^ ^]^

Photodetectors quantify electromagnetic wave information by measuring the changes in electrical conductivity caused by light radiation.^[^ ^15^, ^16^ ^]^ They offer several benefits—such as their small size, lightweight construction, rapid response speed, and high sensitivity—resulting in their widespread use in military applications and various aspects of civilian life.^[^ ^17^, ^18^, ^19^, ^20^ ^]^ Photodetectors can be employed in spectrometers, fluorescence microscopes, laser rangefinders, fiber‐optic communications, LiDAR, and night‐vision devices, amongst others.^[^ ^21^, ^22^, ^23^ ^]^ However, the current use of photodetectors faces considerable challenges such as the need to match the frequency waveform and incident radiation energy of the measured signal, as well as the requirement for integrating complex optical and electronic systems.^[^ ^24^, ^25^ ^]^ Moreover, reducing the size of photodetectors and improving their measurement responsiveness, noise equivalent power, and detection rate is crucial for their continued development.^[^ ^26^, ^27^, ^28^, ^29^ ^]^

The use of metasurfaces in photodetector devices can effectively enhance the performance and overcome certain limitations:^[^ ^30^, ^31^, ^32^ ^]^ 1) Metasurface‐based photodetectors (metaphotodetectors) enable a wider range, greater sensitivity, and faster response, thereby improving their detection performance.^[^ ^33^ ^]^ 2) Metaphotodetectors avoid the need for more complex optical systems, enabling miniaturization, and broadening their application potential.^[^ ^34^ ^]^ 3) Metaphotodetectors can simultaneously perform multiple functions, thereby effectively promoting the development of multimodal full‐sensing integrated systems.^[^ ^35^ ^]^

**Figure 1:**
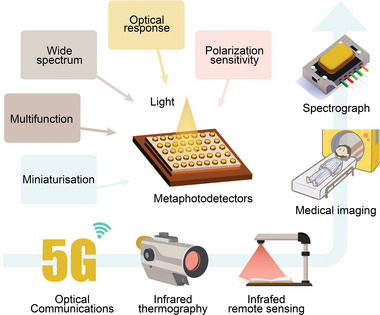
The advantages and applications of metaphotodetectors.

This review provides a comprehensive overview of the advantages of employing metasurfaces in metaphotodetectors, focusing on five aspects: broad spectrum, photo response, polarization sensitivity, miniaturization, and multifunctionality (**Figure** **1**). It extensively explores the applications of metaphotodetectors in various fields including optical communication, infrared thermal imaging, infrared remote sensing, medical imaging, and spectroscopy. Additionally, it discusses the potential impact of metaphotodetectors on human life as well as their future prospects and developmental trends.

## 2 Concepts and Principles of Metasurfaces

### 2.1 Physical Concepts and Properties

Metamaterials are artificially designed materials with unique properties that are not found in natural materials.^[^ ^36^ ^]^ A metasurface is a 2D metamaterial with ultrathin thickness at the wavelength level, which reduces its dimensionality from 3D volume structures to 2D surface structures.^[^ ^37^ ^]^ Composed of multiple sub‐wavelength‐scale repeating units, metasurfaces can be designed to manipulate electromagnetic waves by precisely designing the geometric shapes, sizes, directions, and arrangements of the repeating units.^[^ ^38^ ^]^ Unlike traditional optical elements, which rely on the accumulation of an optical path under the constraints of Snell's law to change the phase, metasurfaces offer a solution to the limitations of large volumes and simple functions.^[^ ^39^ ^]^ They can also effectively overcome the drawbacks of large dispersions in metamaterials, the difficulty in achieving broadband operation, and inevitable internal adsorption losses.^[^ ^40^ ^]^ Consequently, metasurfaces have applications in negative refraction,^[^ ^2^, ^7^, ^11^ ^]^ super lenses,^[^ ^4^, ^14^ ^]^ super‐resolution,^[^ ^1^, ^9^, ^41^ ^]^ and highly directional antennas.^[^ ^3^, ^6^, ^42^ ^]^

### 2.2 Geometric Structure

When designing geometric structures of metasurfaces, it is essential to clarify their functional requirements. The geometric structure can be tailored to achieve specific optical properties through precise design and adjustments. By precisely designing and adjusting the geometric structure, the light propagation direction, wavefront shape, reflectivity, absorption, and dispersion properties can be controlled.^[^ ^43^, ^44^, ^45^ ^]^ Specifically, the design process of metasurfaces should follow the following principles: 1) The design should be based on the functional requirements of metasurfaces, as metasurfaces with different functional requirements have completely different design ideas; 2) Specific types of materials should be selected based on the working environment of the metasurface to ensure the stability and durability of the microstructure during use; 3) Factors such as material properties and operating frequency need to be considered in the design process of metasurface basic units; 4) Numerical simulation and optimization methods should be combined in the process of metasurface design to predict and optimize the performance of metasurfaces, and to obtain the parameters and structure with the best performance; 5) The manufacturing of metasurfaces usually involves micro/nano processing technology, thus process limitations must be considered. 6) The application of metasurfaces generally requires integration with other components or systems, and the ease of integration into systems must also be considered during design.

The Fermat principle states that light travels along a path that minimizes the time between two points. In 2011, Yu et al. proposed generalized laws of refraction and reflection that extended the Fermat principle to metasurfaces.^[^ ^39^ ^]^ They derived laws governing the relationship between incoming and outgoing waves, as well as reflected waves:

(1) $$n_{t}\;Sin\left(\theta_{t}\right)-n_{i}\;Sin\left(\theta_{i}\right)=\frac{\lambda_{0}}{2\pi }\frac{d\phi \left(\chi \right)}{d\chi }$$

(2) $$n_{i}\;Sin\left(\theta_{r}\right)-n_{i}\;Sin\left(\theta_{i}\right)=\frac{\lambda_{0}}{2\pi }\frac{d\phi \left(\chi \right)}{d\chi }$$

where *n_i_* and *n_t_* represent the refractive indices on the two sides of the interface, *λ*_0_ is the free space wavelength, *θ_i_*, *θ_r_*, and *θ_t_* are the incident angles, reflected angles, and transmitted angles, and *d_ϕ_/d_x_* indicates the gradient of the phase discontinuity along with the interface, provided by the metaatoms of the metasurface.

Various types of metasurfaces have been designed on the basis of these principles and control equations. Common metasurface geometries include metal nanopillars, nanopores, and 2D periodic structures. The different geometric structures mentioned above often follow different principles. Metal nanopillars can serve as guiding structures for electromagnetic waves. The phase delay of electromagnetic waves passing through metasurfaces can be adjusted by changing the height, spacing, and arrangement of the pillars, thereby achieving phase‐gradient changes for beamforming or wavefront control. Metal nanopores have a unique ability to regulate the transmission, reflection, and scattering characteristics of electromagnetic waves, which are influenced by the pore size and shape. At the same time, the frequency and intensity of metal surface plasmon resonance also can be controlled by adjusting the size, shape, and arrangement of nanopores. The principle of 2D periodic structured metasurfaces mainly involves the control of the beam by the size and shape of the microstructures, and the interference and diffraction effects of periodic characteristics on electromagnetic waves. For example, Bragg diffraction modulates the beam, the Brillouin zone precisely controls the beam, and the photonic crystal effect controls the reflection or transmission of electromagnetic waves within a specific wavelength range. **Table** **1** summarizes the applications of metasurfaces of different structural types.

**Table 1:** The applications of metasurfaces with different structural types.

Structural Type	Material	Application	Ref.
Dielectric nanopore array	Ge	Improved responsivity and speed of the photodetectors at 2 µm wavelengths	[52]
Plasmonic metasurface square cavities	–	Next‐generation quantum well photodetectors with more complex optical response	[53]
Omega‐shaped optical nanoantenna	Au	Broadband and tunable absorption peaks at 1550 nm communication wavelengths	[54]
Fractal metasurface with the morphology of snowflakes	Au	Enabling broadband and polarisation‐insensitive optoelectronic enhancement	[55]
Phase‐gradient metasurfaces	Au	Broadband enhancement of the efficiency of organic photodiodes	[56]
Disc‐shaped plasma metasurface	Au	Black phosphorus‐based mid‐infrared photodetector	[57]
Modified cross‐resonator structures	Colloidal quantum dots	Highly responsive and compatible with CMOS circuits in the 1550 nm wavelength range	[58]
Gap surface plasmon polariton	MoS_2_	Development of high‐performance photodetectors with high polarisation sensitivity and fast response	[62]
Chiral plasmonic metasurfaces	Au	Accurate extraction of the full state of polarization (SoP) information of 1550 nm infrared polarized light	[63]
Helical nano‐antenna array	Al	Distinguishing linearly polarised light from circularly polarised light in quadrature without additional filters	[65]
Anisotropic antenna array	Hybrid organic–inorganic perovskite (HOIP)	Detect both linearly polarized and circularly polarized light simultaneously	[66]
Grating composed of scatterers arranged in a subwavelength periodic	Silicon nitride	High transmittance and excellent beam control characteristics	[67]
Nanostripe array	Au	Unique sensitivity to the local direction of light propagation, enabling phase contrast detection below 10 mrd	[69]
Nanopore array metasurfaces	–	Directly distinguish between the direction of rotation and photocurrent of circularly polarised light	[70]
Anisotropic metasurfaces	Au	Antennas for wearable devices	[72]
Nanopillar array	Si	Focal length adjustment from 600 to 1400 µm at an operating wavelength of 915 nm	[73]
First‐order cross fractal	Phase‐changing material vanadium dioxide (VO_2_)	Realization of broadband absorption	[74]

Ref.: Reference

### 2.3 Principles of Optical Manipulation

Metasurfaces are typically composed of periodic microstructural units, with dimensions significantly smaller than the wavelength of light. This enables the precise control and manipulation of light.^[^ ^46^, ^47^ ^]^ This principle involves two components: reflection and transmission. By designing microstructural units with specific phase and amplitude responses, we can manipulate light reflection and transmission.^[^ ^48^ ^]^ The size, shape, and arrangement of the metasurface microstructural units can change the amplitude, phase, and propagation direction of the reflected light. This effectively allows operations such as focusing, deflection, and polarization conversion. On the other hand, the manipulation of phase modulation, beam focusing, and changes in diffracted light for transmitted light is achieved through the precise design of parameters for metasurface microstructure unites. This allows control over the intensity, direction, and phase of the transmitted light. The principle of optical manipulation of metasurfaces can be explained using electromagnetic and diffraction theories.^[^ ^49^ ^]^ The response of the microstructural units to light is closely related to their size, shape, and material properties.^[^ ^50^, ^51^ ^]^ By precisely designing these parameters, we can achieve precise light control, thereby enabling various optical manipulation functions.

## 3 Application of Metasurfaces in Metaphotodetectors

### 3.1 Broad Spectrum

Photodetectors play a vital role in various fields such as industry, daily life, and military applications. As the complexity of photoelectric detection continues to increase, it is necessary to integrate photodetectors operating in different bands to achieve broadband detection in the same scene. However, conventional broadband detection often requires multiple detectors from different bands to operate together, leading to an increase in system complexity. Therefore, the development of ultra‐wideband photodetectors capable of broad‐spectrum detection has become a cutting‐edge research topic worldwide. One of the challenges in achieving broadband detection is the limited detection range of traditional semiconductor photodetectors, which is constrained by the semiconductor bandgap. To overcome this limitation, metasurfaces with unique electromagnetic wave manipulation capabilities have been explored. Metasurfaces exhibit excellent design flexibility and can achieve combinations of various microstructures. By adjusting the size, shape, arrangement, and other parameters of the microstructures, they can respond to a wide spectral range from visible light to microwaves. By combining different types of microstructures, it is possible to simultaneously respond to multiple wavelength ranges, thereby achieving a wide spectral performance. By incorporating metasurfaces into photodetectors, it becomes possible to effectively expand their detection range.

**Figure 2:**
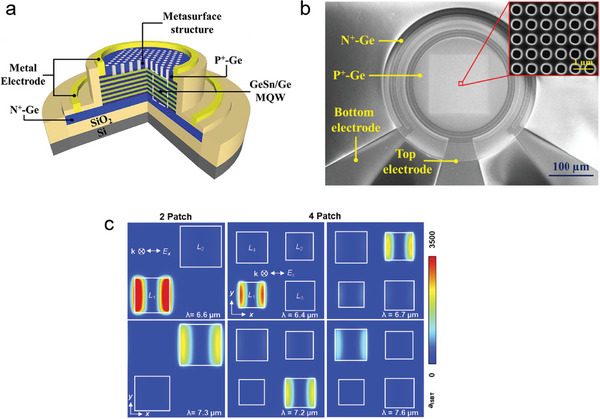
a) Schematic diagram of the GeSn/Ge MQW photodetector with a dielectric nanohole array metasurface.^[^ ^52^ ^]^ Copyright 2023, American Chemical Society. b) The SEM top‐view image of GeSn/Ge MQW photodetector (inset: magnified view of the metasurface).^[^ ^52^ ^]^ Copyright 2023, American Chemical Society. c) The local ISBT absorbance within quantum well layer *a*_ISBT_ (1 µm^−2^) at the resonant wavelength of a single patch in the detector.^[^ ^53^ ^]^ Copyright 2020, Institute of Electrical and Electronics Engineers.

The development of short‐wavelength infrared photodetectors that can meet the increasing capacity of single‐mode fiber optic communication systems is of great significance as these systems approach their theoretical capacity limit. Currently, the commonly used shortwave infrared photodetectors are based on III–V group materials, II–VI group materials, and 2D materials. However, these photodetectors often suffer from high cost and incompatibility with complementary metal–oxide–semiconductor (CMOS) technology, and their performance in terms of detection accuracy and other aspects is still unsatisfactory. Chen et al. have successfully constructed a GeSn/Ge multiple quantum well (MQW) photodetector with a dielectric nanopore array metasurface, which effectively improves the low‐cost detection of short‐wavelength infrared light and achieves higher detection accuracy (**Figure** **2**a,b).^[^ ^52^ ^]^ By optimizing the metasurface structure, the responsivity of the photodetector at 2 µm wavelengths can be increased by 10.5 times. Compared with traditional GeSn/Ge MQW photodetectors, the 3D bandwidth has been increased by 35%. Theoretical predictions suggest that this photodetector can achieve room temperature detection performance comparable to current commercial shortwave infrared photodetectors. This contribution validates the possibility of achieving compatibility between high‐performance optoelectronic detection and CMOS technology and provides a new approach for achieving low‐cost and efficient optoelectronic detection in the 2 µm communication band.

Quantum well‐infrared photodetectors (QWIPs) have attracted considerable attention due to their high sensitivity and excellent signal‐to‐noise ratio. However, the absorption range of traditional QWIPs is limited as they can only detect optical signals through sub‐band transitions (ISBTs) between conductive bands, which restricts their ability to absorb polarized light perpendicular to the surface of the quantum well. To address this limitation, researchers have combined QWIPs with plasma cavities to enhance absorption efficiency through electromagnetic field enhancement and polarization rotation within the cavity. However, this approach still imposes limitations on the absorption range of the detector. In a recent study, Hainey et al. successfully expanded the spectral response range of quantum well photodetectors by 1.5 times while maintaining high detection performance by splicing different resonant cavities within a single sub‐wavelength period (Figure 2c).^[^ ^53^ ^]^ This achievement holds great significance for the development of next‐generation quantum well photodetectors with more complex optical responses.

### 3.2 Photo Response

The light response capability of photodetectors is a crucial indicator for evaluating their performance. Currently, researchers have investigated photodetectors based on various materials including graphene, organic semiconductors, black phosphorus, and colloidal quantum dots. Although these materials have demonstrated unique advantages, their light response performance remains unsatisfactory. By employing metasurface technology, the photoresponse ability of photodetectors based on the aforementioned materials has been effectively enhanced.

**Figure 3:**
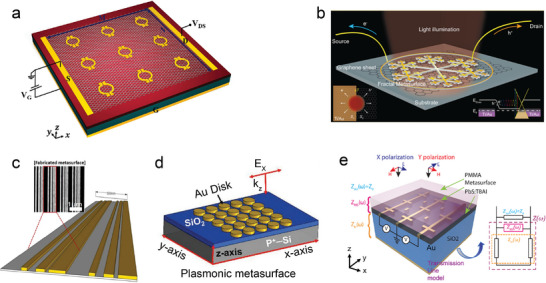
a) Schematic diagram of a graphene photodetector with an omega‐shaped nanoantenna array modified with Au on a single‐layer graphene.^[^ ^54^ ^]^ Copyright 2021, Elsevier. b) Schematic diagram of the structure and principle of a graphene photodetector integrated with a fractal tree metasurface with snowflake geometry.^[^ ^55^ ^]^ Copyright 2016, American Chemical Society. c) Schematic diagram of a 3D model of a metasurface (inset: SEM image of metasurface).^[^ ^56^ ^]^ Copyright 2018, American Chemical Society. d) Schematic diagram of plasma element surface based on Au disk.^[^ ^57^ ^]^ Copyright 2023, John Wiley and Sons. e) Schematic diagram of metasurface enhanced photodetectors based on colloidal quantum dots and their compatibility with CMOS circuits.^[^ ^58^ ^]^ Copyright 2022, American Chemical Society.

Graphene and other 2D materials possess numerous advantages, including wide spectral absorption, high carrier mobility, and ultrafast time response, which make them ideal options for photodetectors. However, the limited light absorption ability of these materials somewhat restricts their application in the field of photodetectors. Attarabad et al. fabricated a graphene photodetector integrated with a plasma metasurface, effectively overcoming the limitation of photodetectors caused by the insufficient light absorption capacity of graphene (**Figure** **3**a).^[^ ^54^ ^]^ The metasurface consists of an Omega‐shaped optical nanoantenna, which enhances the interaction between light and graphene, significantly improving the light absorption capability. By manipulating the geometric parameters of the metasurface, broadband, and tunable absorption peaks are achieved at a communication wavelength of 1550 nm, with the absorption power in graphene increased to 66%. This photodetector exhibits promising applications in areas such as nanosensing, imaging, optical communication, and biomedical monitoring. Fang et al. fabricated a fractal metasurface by replicating the geometric morphology of snowflakes using a fractal tree structure. They then integrated this fractal metasurface onto graphene photodetectors (Figure 3b).^[^ ^55^ ^]^ By exciting plasmon oscillations at the interface between graphene and metal, they achieved localized enhancement of electromagnetic waves, leading to the generation of a large number of electron–hole pairs in graphene. The spatial separation of these pairs is achieved through the built‐in electric field (PV effect) and the difference in thermoelectric power (PTE effect), resulting in the generation of detectable photovoltage. This approach enables broadband and polarization‐insensitive photoelectric enhancement, with an enhancement factor ranging from 8 to 13 in the visible spectrum. Furthermore, this strategy can also be extended to integrate with other PV/PTE materials, offering good flexibility.

Organic photodetectors possess advantages such as portability, low cost, and the capability for large‐scale production. They have found wide applications in fields such as photoelectric conversion and energy harvesting. However, one limitation is their restricted absorption capacity for specific wavelengths of light due to the materials themselves. To address this issue, researchers have employed various techniques such as Fabry Perot cavities, plasma structures, and photonic crystal architectures to enhance light absorption. Xu et al. integrated a metasurface with broadband functionality into organic photodetectors, resulting in a significant improvement in light absorption and photocurrent generation within the wavelength range of 560–690 nm (Figure 3c). This improvement led to a 1.5 to 2 times enhancement in response performance, thanks to the broadband characteristics of the metasurface.^[^ ^56^ ^]^ This study holds great significance in enhancing the performance of organic photodetectors.

Black phosphorus (BP) is a van der Waals semiconductor material with directly adjustable bandgap and anisotropic optical properties, making it an ideal choice for manufacturing mid‐infrared (MIR) photodetectors. However, the small optical absorption cross‐section of BP at room temperature limits the performance of current BP‐based MIR photodetectors, making it challenging to achieve the ideal combination of gain and response time. Yadav et al. designed and fabricated a plasma metasurface based on Au disks (Figure 3d), which exhibits localized surface plasmon resonance (LSPR) in the MIR region (3.7 µm) and can match the band edge of BP.^[^ ^57^ ^]^ By integrating BP sheets onto the plasma metasurface, they successfully achieved quenching of photoluminescence, thus creating a BP‐based MIR photodetector that effectively achieved a peak response of 495.85 mA W^−1^ and an ultrahigh operating speed (>10 MHz) at an incident wavelength of 3.7 µm. This opens up new opportunities for optoelectronic applications in the MIR region.

Colloidal quantum dots (QDs) possess tunable bandgaps and the potential for low‐cost solution processing, making them an economically viable alternative to III–V semiconductor technology. However, achieving high responsiveness and low noise equivalent power, as well as their utilization in a wide range of spectral windows, remain challenging in their development. Leuthold et al. developed a metasurface‐enhanced photodetector based on colloidal quantum dots (Figure 3e), which achieved a response of up to 8000 A W^−1^ in the wavelength range of 1550 nm, demonstrated a noise equivalent power as low as tens of picowatts per hertz, and achieved compatibility with CMOS circuits.^[^ ^58^ ^]^ The high responsiveness is attributed to the successful application of a metasurface that increases the absorbance by ten times that of quantum dot films of the same thickness, achieving optical gain. This work not only enhances device performance but also provides new solutions for future optoelectronic integrated systems.

Silicon‐based detectors are commonly used for extracting optical image information, and the optical response characteristics of these detectors limit their ability to completely extract optical information stored in different degrees of freedom. In real life, we often need to extract image information in complex environments. In order to achieve complete information extraction, silicon‐based detectors always require additional optical components and multiple measurements and are often affected by environmental interference, leading to a decrease in imaging quality. Xiong et al. designed and manufactured a dual‐layered integrated perovskite single‐pixel detector (DIP‐SPD), which has the ability to recognize stacked dual‐color metasurface images in complex environments. This work provides new ideas for the extraction of color images in metasurfaces and paves the way for the application of perovskite materials in color imaging.^[^ ^59^ ^]^

### 3.3 Polarization Sensitivity

The phenomenon of the spatial distribution of optical wave electric vector vibration losing symmetry in the direction of light propagation is called polarization of light. The polarization state of light can provide rich information such as shape, texture, direction, or strain. Therefore, accurately measuring the polarization state of light is of great significance, which increases the demand for photodetectors with polarization measurement capabilities. Traditional polarization measurement methods generally require complex optical systems, and metasurface technology can achieve polarization selective absorption, transmission, and beam guidance through its special surface structure. Some cutting edge literatures on the sensitivity of metasurface plarization detection are listed in **Table** **2**.

**Figure 4:**
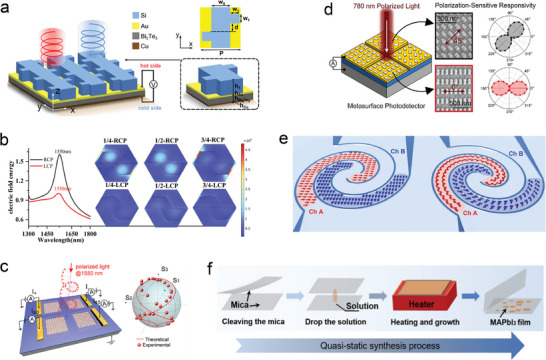
a) Schematic diagram of the chiral photodetector based on the photothermoelectric effect.^[^ ^60^ ^]^ Copyright 2023, Optica Publishing Group. b) Space volume electric field integration in amorphous silicon based on six small pixels in the surface of all Stokes large pixel cells.^[^ ^61^ ^]^ Copyright 2023, Optica Publishing Group. c) Schematic diagram of a chiral plasma metasurface graphene silicon photodetector with a four‐quadrant detector architecture.^[^ ^63^ ^]^ Copyright 2020, American Chemical Society. d) Schematic diagram of ultrathin pyroelectric photodetector structure with integrated polarization sensing metasurface.^[^ ^64^ ^]^ Copyright 2023, American Chemical Society. e) Schematic diagram of two spiral MSM photodetectors with rectangular and chiral nanoantennas.^[^ ^65^ ^]^ Copyright 2022, Optical Society of America. f) Schematic diagram of quasi‐static solution growth process for HOIP thin films used in multi‐polarization sensitive photodetectors.^[^ ^66^ ^]^ Copyright 2016, American Chemical Society.

Hu et al. developed a mid‐infrared circularly polarized photodetector based on chiral metasurfaces and photo thermoelectric effects (**Figure** **4**a). This photodetector can specifically and sensitively detect circularly polarized light and has shown potential in applications such as chiral imaging and chiral molecular detection.^[^ ^60^ ^]^ Cheng et al. simulated and analyzed the influence of geometric parameters of metasurfaces on the absorption of orthogonally polarized light using COMSOL Multiphysics software (Figure 4b).^[^ ^61^ ^]^ They proposed a fully Stokes large pixel based on six small pixels and integrated this metasurface into a nGaAs/InP photodetector. This photodetector can accurately measure any polarized light at a working wavelength of 1550 nm, which is of great theoretical and practical significance for the development of polarization detection technology. Ni et al. developed a metasurface photodetector for gap surface plasmon polariton (GSP) based on the 2D material MoS_2_.^[^ ^62^ ^]^ This research group designed a novel GSP metasurface structure that matches the GSP mode with the neutral A‐exciton resonance of the MoS_2_ monolayer by adjusting the width of the metal nanobarns. This selectively enhances the interaction between light and the MoS_2_ monolayer, resulting in a polarization ratio of ≈0.94. The 2D material‐based photodetector developed in this work has the advantages of high polarization sensitivity and fast response speed, providing a new direction for the design and manufacturing of high‐performance photodetectors applied in fields such as communication, imaging, and sensing. The aforementioned research has effectively enhanced the accuracy of polarization measurement by utilizing metasurface materials. However, these studies necessitate the utilization of external cameras or photodiodes and rely on an external power supply.

Li et al. developed a graphene–silicon photodetector that integrates chiral plasmonic metasurfaces, enabling accurate extraction of the full state of polarization (SoP) information of 1550 nm infrared polarized light (Figure 4c).^[^ ^63^ ^]^ The design of the micro pixels combines the most cost‐effective architecture of four‐quadrant detectors, effectively simplifying the polarization measurement system and enabling analysis of the light's SoP at a resolution of several micrometers. This ingenious design has demonstrated enormous potential in applications such as polarization imaging and chemical sensing. Wilson et al. developed an ultrathin pyroelectric photodetector with an integrated polarization sensing metasurface by utilizing nanoscale metal metasurface structures and the synergistic effect of metasurfaces and pyroelectric structures (Figure 4d).^[^ ^64^ ^]^ This achievement enables high‐sensitivity polarization state detection without the need for external phase machines and power sources. The developed ultrathin pyroelectric detector possesses highly integrated characteristics, providing an effective solution for future optical detection instruments that require high integration and miniaturization. These studies utilize metasurfaces to accurately detect polarized light. However, these photodetectors are inevitably affected by metal loss.

In light of this, Panchenko et al. have developed a novel plasma metasurface differential photodetector by integrating antennas sensitive to orthogonal polarization into two back‐to‐back Schottky photodetectors (Figure 4e).^[^ ^65^ ^]^ The photodetector is capable of distinguishing between linearly and circularly polarized light in orthogonal states without the need for additional filters. It exhibits high sensitivity within the wavelength range of 500 to 800 nm. This new type of photodetector is compatible with traditional CMOS manufacturing processes and is well‐suited for integration into modern optical systems. Li et al. have developed a multi‐polarization sensitive photodetector using a hybrid organic–inorganic perovskite (HOIP) metasurface with a high refractive index and excellent electrical properties. This photodetector can effectively detect both linearly polarized and circularly polarized light simultaneously (Figure 4f).^[^ ^66^ ^]^ This research presents new possibilities for the integration of optoelectronics applications.

**Table 2:** The progress in sensitivity of polarization detection using metasurfaces.

Polarization type	Material	Application	Ref.
Circular polarization	Bi_2_Te_3_	Chiral imaging and chiral molecular detection	[60]
Orthogonal polarization	Silicon	All Stokes polarized light detector	[61]
Unrestricted	Au	Metasurface photodetector for gap surface plasmon polariton (GSP)	[62]
Full state of polarization (SoP)	Graphene–silicon	Polarization imaging and chemical sensing	[63]
Unrestricted	Ag	Ultrathin pyroelectric photodetector	[64]
Linear polarization or circular polarization	Al	Compact, CMOS compatible plasma metasurface photodetector	[65]
Linear polarization or circular polarization	Hybrid organic–inorganic perovskite (HOIP)	Efficient polarization photoelectric detection	[66]

Ref.: Reference

### 3.4 Miniaturization

**Figure 5:**
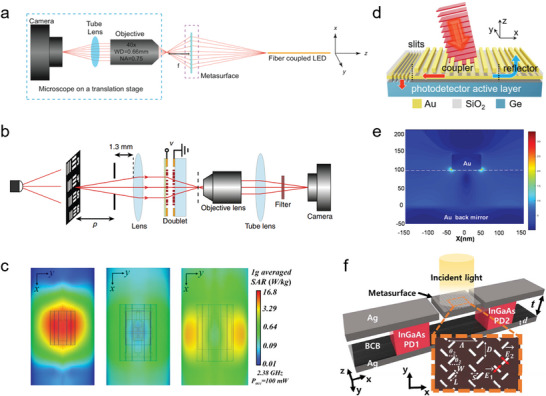
a) A microscope setup model image that can be translated along the optical axis for focal plane imaging.^[^ ^67^ ^]^ Copyright 2016, American Chemical Society. b) Schematic diagram of using an adjustable frequency multiplier for imaging.^[^ ^71^ ^]^ Copyright 2018, Nature Publishing Group. c) Simulated 1 g averaged SAR values for the planar monopole, the integrated metasurface‐enabled antenna, and the reference patch antenna (1 nm away from the multilayer organizational model).^[^ ^72^ ^]^ Copyright 2014, Institute of Electrical and Electronics Engineers. d) Schematic diagram of the device structure and working principle of asymmetric metasurface photodetectors.^[^ ^69^ ^]^ Copyright 2023, the author(s), published by De Gruyter, Berlin/Boston. e) The calculation results of the electric field intensity in the plane transverse to the metal stripe.^[^ ^62^ ^]^ Copyright 2019, IOP Publishing Ltd. f) Schematic diagram of CPA with 2D metasurfaces and embedded photodetectors.^[^ ^70^ ^]^ Copyright 2018, Institute of Electrical and Electronics Engineers.

With the continuous advancement of optical technology, the demand for miniaturization is increasing. Metasurfaces are composed of 2D quasi‐periodic arrays with subwavelength structures. Their unique structure can provide discrete phase changes for incident light, eliminating the need for gradual phase accumulation through light propagation, thus facilitating the miniaturization of optical devices.^[^ ^67^, ^68^ ^]^ Zhang et al. proposed a method for producing high‐quality metasurface optical elements with low loss and high numerical aperture (NA≈0.75) using silicon nitride (**Figure** **5**a).^[^ ^67^ ^]^ These elements demonstrate high transmittance and excellent beam control characteristics in the visible light range and can be integrated with both silicon detectors and traditional CMOS manufacturing technologies. Ni et al. developed a hybrid metasurface photodetector incorporating a MoS_2_ gap mode and metal nanostrip arrays on MoS_2_ layers (Figure 5b).^[^ ^62^ ^]^ In this structure, each metal Au nanostrip acts as an individual interstitial plasma cavity, selectively enhancing the interaction between light and matter within a single‐layer MoS_2_. This effectively improves the polarization sensitivity and response speed of the photodetector. The study demonstrates the potential for controlling advanced performance through strong electromagnetic field confinement and resonant coupling between exciton resonance and gap plasma resonance. It also highlights the use of integrated optical metasurfaces as a novel approach to enhance the performance of photodetectors based on 2D materials, offering great potential for miniaturization and performance improvement.

Traditional image sensors can only capture the intensity distribution of incident light and cannot obtain phase information. To overcome this limitation, additional components are often required, making the system bulky and hindering the development of technologies such as biomedical microscopes. In a recent study, Liu et al. introduced a novel photodetector coated with a specially designed plasma metasurface (Figure 5c).^[^ ^69^ ^]^ This photodetector is sensitive to the local propagation direction of incident light and can capture both the intensity and phase information without the need for any additional optical components. This breakthrough simplifies phase imaging systems and improves imaging speed, which has significant implications for the miniaturization of biomedical microscopes and other equipment.

In addition, as mentioned earlier, chiral discrimination of circularly polarized light (CPL) is crucial for various application scenarios. However, traditional methods for CPL discrimination rely on bulky optical components. In recent years, metasurfaces have emerged as a potential solution to this problem. However, most research still focuses on passive devices with only a single metal–dielectric interface. To address this limitation, Park et al. proposed a novel structure based on the Metal Dielectric Metal (MDM) platform for an ultrathin circular polarization analyzer (CPA) (Figure 5d).^[^ ^70^ ^]^ The analyzer integrates nanopore array metasurfaces and semiconductor photodetectors to directly distinguish the rotation direction of circularly polarized light from photocurrent, eliminating the need for traditional optical components.

Traditional zoom lenses typically require changing the axial distance between multiple optical components in order to achieve zoom, which limits their ability to zoom within a small range and necessitates a large lens volume. Arbabi et al. developed an ultrathin tunable focal length metasurface dual lens based on microelectromechanical systems (MEMS) (Figure 5e).^[^ ^71^ ^]^ This metasurface can achieve an optical power change of 60 diodes by moving 1 µm, with a scanning frequency that can reach several thousand hertz. The system demonstrates optical power adjustment capability of over 180 diopters and achieves a focusing efficiency of over 40%. Additionally, it can be further integrated with a third metasurface to produce a compact microscope with a thickness of only 1 mm. The design of this MEMS‐integrated metasurface provides a new platform for integrating tunable and reconfigurable optical systems, which in turn promotes the miniaturization of optical components. It also opens up more innovative possibilities for the development of compact, fast‐scanning endoscopes, confocal microscopes installed at fiber tips, and other devices.

With the rapid development of medical technology, wearable medical devices are playing an increasingly important role in daily diagnosis and treatment. Therefore, it is necessary to develop and design antennas that have high radiation efficiency, low profile, lightweight, and minimal impact on human tissues to meet the needs of wearable medical devices. Jiang et al. have developed a novel wearable antenna using highly truncated anisotropic metasurfaces (Figure 5f).^[^ ^72^ ^]^ This antenna has a small volume and can achieve an impedance bandwidth of 5.5%, a gain of 6.2 dBi, and a front‐to‐back ratio of over 23 dB in the medical body area network (MBAN) frequency band of 2.36–2.4 GHz. Even under extreme deformation conditions, the antenna can still cover a reasonable bandwidth and maintain a good gain and front‐to‐back ratio. Moreover, this antenna can ensure low absorption of electromagnetic energy in human tissues, making it an ideal choice for future wearable medical device applications.

### 3.5 Multifunctional

**Figure 6:**
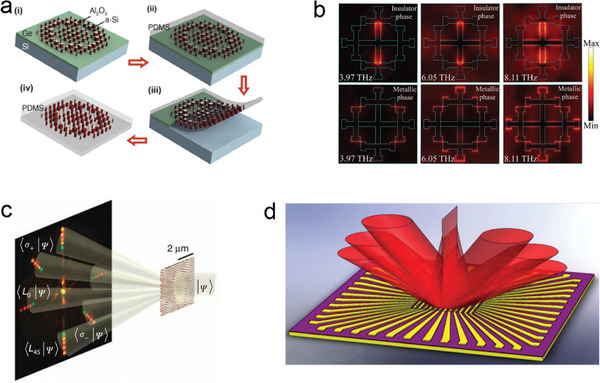
a) Manufacturing steps and device images of highly adjustable dielectric metasurface lenses based on silicon nanopillars and transparent elastic polymer packaging.^[^ ^73^ ^]^ Copyright 2016, John Wiley and Sons. b) The electric field distribution at the interface between the mixed resonator and the dielectric spacer when VO_2_ is in an insulating state at different frequencies and after IMT.^[^ ^74^ ^]^ Copyright 2022, IOP Publishing Ltd. c) Schematic diagram of a silicon‐based SPM illuminated by an elliptical polarized multicolor light source.^[^ ^75^ ^]^ Copyright 2017, The Author(s). d) Schematic diagram of a multifunctional metasurface with 96 independently addressable metasurface elements.^[^ ^76^ ^]^ Copyright 2022, American Chemical Society.

Ultrathin optical elements are of great significance for the development of highly integrated and miniaturized electronic devices. Numerous studies have demonstrated the exceptional potential of metasurfaces in high‐performance electronic devices. However, most tunable metasurface elements typically rely on diffraction and plasmon effects, which limit the tuning range. Kamili et al. developed a highly tunable dielectric metasurface lens based on silicon nanopillars and transparent elastic polymer packaging (**Figure** **6**a). This lens can achieve focal length adjustment from 600 to 1400 µm at a working wavelength of 915 nm, while still maintaining diffraction‐limited focusing and a focusing efficiency above 50%.^[^ ^73^ ^]^ The design and manufacturing of this tunable metasurface lens provide a direction for the development of ultrathin, multifunctional, and tunable optical devices. Badri proposed a simple configuration for a multifunctional and switchable terahertz metasurface absorber, which is insensitive to polarization and based on the phase‐changing material vanadium dioxide (VO_2_) (Figure 6b).^[^ ^74^ ^]^ By connecting VO_2_ patches to the first‐order cross fractal of gold, the absorber can switch from a narrowband absorber to a broadband absorber, effectively converting the resonator into a third‐order cross fractal. In the insulating phase of VO_2_, the main narrowband absorption occurs at a frequency of 6.05 THz, with an absorption rate of 0.99 and a half‐width at half maximum (FWHM) of 0.35 THz. During the transition from the insulating phase to the metallic phase of VO_2_, the metasurface achieves broadband absorption with an FWHM of 6.17 THz.

In recent years, the continuous development of electronic science and technology has led to widespread attention on the development of multimodal full‐sensing integrated systems. Therefore, the development of optical systems capable of performing multiple functions simultaneously is of great significance. In the field of optics, the utilization of common aperture geometric phase metasurfaces (GPM) through interleaved nanoantenna arrays not only enables precise control of optical wavefronts but also facilitates the realization of multiple functions on a single plane. Maguid et al. have developed silicon‐based devices consisting of multiplexed geometric phase profiles and investigated the performance limitations of interleaved nanoantenna arrays to determine the ultimate information capacity of the photonic system (Figure 6c).^[^ ^75^ ^]^ This research lays a solid foundation for the development of multimodal real‐time detection devices integrated into chips. Shirmanesh et al. developed a multifunctional programmable metasurface prototype capable of dynamically steering beams and reconfiguring light focusing through electro–optic control of the phase of scattered light in each unit of the metasurface (Figure 6d).^[^ ^76^ ^]^ This compact, lightweight, and stationary electronic control array enables the achievement of multiple optical functions within the telecommunications wavelength range. By programming and controlling the phase, amplitude, and polarization of scattered light in the near‐infrared wavelength range, various optical functions can be achieved. As a conceptual validation, the design demonstrates the utilization of the same device for both beam steering and dynamic focusing by designing phase profiles for the multifunctional element surface. Furthermore, through algorithm optimization and the integration of electro–optical devices on chips, the efficiency of the multifunctional element surface can be improved to develop fast and energy‐efficient programmable devices.

## 4 Practical Application of Metaphotodetectors

### 4.1 Optical Communication

**Figure 7:**
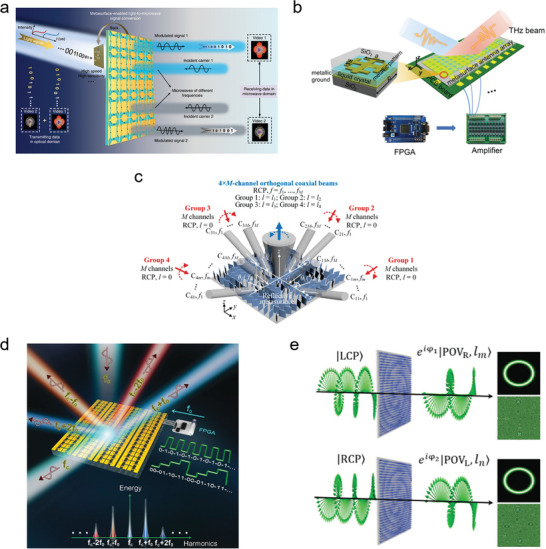
a) Schematic diagram of direct light‐to‐microwave signal conversion using a hybrid time‐varying metasurface platform.^[^ ^77^ ^]^ Copyright 2022, The Author(s). b) Schematic diagram of the THz programmable metasurface.^[^ ^78^ ^]^ Copyright 2020, The Author(s). c) A Schematic diagram of 2D multichannel multiplexing combined with spatial and frequency domains has been achieved through a reconfigurable dielectric element surface operating in reflection and transmission modes.^[^ ^79^ ^]^ Copyright 2022, The Author(s). d) Schematic diagram of time domain digital coding element surface.^[^ ^80^ ^]^ Copyright 2018, The Author(s), Published by Oxford University Press on behalf of China Science Publishing & Media Ltd. e) Schematic diagram of the metasurface that can provide two independent phase profiles for LCP and RCP light, as well as the intensity and phase profiles of the POV generated by the metasurface with lm = 5 and ln = 10.^[^ ^81^ ^]^ Copyright 2021, Nature Publishing Group.

The signal conversion process in traditional wireless communication technologies typically requires complex relay systems, resulting in large volumes and high costs. It is important to effectively integrate optical communication with microwave communication technology to achieve more efficient and secure data transmission. Currently, no equipment is available for encoding optical signals onto microwave carriers. Metasurfaces are 2D artificial structures that can manipulate multiple electromagnetic waves by regulating their microstructure, enabling the simultaneous processing of optical and radio frequency signals. Zhang et al. designed and manufactured a metasurface light‐microwave converter with visible light control by integrating a microsecond‐level photoelectric detection circuit onto the surface of a microwave element (**Figure** **7**a).^[^ ^77^ ^]^ This converter can directly convert light signals to microwave signals. The constructed dual‐channel hybrid wireless communication system can independently transmit two different videos simultaneously. This design reduces the need for relay systems and enables dual‐channel data transmission based on frequency division multiplexing, thereby enhancing the information‐processing capabilities. This study demonstrates excellent prospects for the future of wireless communication. This is achieved by encoding optical signals onto microwave carriers and effectively combining optical and microwave communication technologies for more efficient and secure data transmission.

The terahertz band used in the upcoming 6G technology exhibits unique advantages and enormous application potential owing to its high data transmission rate potential, with the potential to achieve high data transmission rates at the Tbit/s level. However, the high propagation loss attenuation problem of terahertz waves in the atmosphere limits their application in the field of communication. To address this challenge, researchers have committed to developing a beamforming technology that can dynamically and directionally propagate terahertz waves. Wu et al. introduced a liquid‐crystal‐based terahertz programming metasurface, which is a linear array of 24 elements (Figure 7b).^[^ ^78^ ^]^ Each element consists of 50 rows and 2 columns of cells. Each cell can achieve an electrically controlled phase change in a 1D array and a maximum THz beam deflection angle of 32° through the designed encoding sequence. This study demonstrates the potential application of liquid‐crystal‐programmed metasurfaces in terahertz beamforming and provides a new approach for active intelligent beamforming in the field of terahertz communication. In addition, vanadium dioxide (VO₂) demonstrates excellent switching behavior from an insulating state to a metallic state in the terahertz region. By controlling the metallic or insulating state of VO₂ bound to the metasurface, a reconfigurable metasurface can be achieved, allowing for dynamic adjustment of the electromagnetic properties. Wang et al. developed a method based on VO₂ that utilizes a novel hybrid terahertz dielectric metasurface composed of 14 cells (Figure 7c).^[^ ^79^ ^]^ This metasurface enables 2D multichannel multiplexing in both reflection and transmission modes across two distinct frequency bands. Simultaneous polarization control and adjustment of the operating frequency band can be achieved by switching the state of VO₂. The above studies provide strong support for the application of metasurfaces in 6G technology.

The contributions of metasurfaces mentioned above provide new possibilities for optical communication technology. However, there is a bottleneck problem regarding the frequency conversion efficiency, which falls short of meeting the practical application requirements under weak signals. Zhao et al. introduced a dynamic time‐domain digital encoding metasurface that effectively manipulates the distribution of spectral harmonics (Figure 7d).^[^ ^80^ ^]^ This manipulation enables the generation and control of nonlinear responses in free space. By controlling the discrete reflection phase state of the metasurface through a digital encoding sequence, they achieved time modulation of the incident wave. This modulation accurately controls the amplitude and phase distribution of all harmonics. Consequently, they have developed a new wireless communication system architecture that not only maintains excellent real‐time signal transmission performance but also greatly simplifies it. This work has made valuable experimental explorations toward the simplification of future practical communication system structures.

The Poincaré beam is a type of beam that has spatially varying polarization. It carries both spin angular momentum (SAM) and orbital angular momentum (OAM), making it advantageous in various fields such as optical communication. However, the generation of traditional Poincaré beams relies on complex optical components and their radius depends on the number of topological charges, which greatly limits their application range. Liu et al. have designed an all‐dielectric metasurface using titanium dioxide (TiO_2_) nanorods spaced at subwavelength distances (Figure 7e).^[^ ^81^ ^]^ This metasurface can achieve all states of perfect Poincaré beams (PPB) on HyOPS without the need for any additional optical components. It enables the broadband generation of generalized PPB without limitations on the topological charge through unique phase modulation. This study presents a new solution for the compact and efficient generation of PPB in the visible light range. It also provides new ideas for the design of future optical systems.

The aforementioned research has made significant advancements in the utilization of metasurfaces in optical communication. However, it has yet to achieve a simultaneous combination of large turning angles, multiple channels, reconfigurability, and ultra‐compactness. This limitation has restricted the application scope of Optical Wireless Communication technology. Tao et al. have developed a compact full‐duplex optical wireless broadcasting communication system utilizing a metasurface measuring only 2 × 2 mm.^[^ ^82^ ^]^ The system can achieve a beam turning angle of up to ±40°, with downlink and uplink capacities of 100 and 10 Gbps per user channel, respectively. Additionally, it offers three operational modes for flexible signal switching by altering the polarization state of the beam. It is noteworthy that these beam‐turning metasurfaces are mass‐produced using complementary metal–oxide–semiconductor process platforms, showcasing the potential for widespread commercial application and presenting novel solutions for future optical communication technologies. Based on this foundation, the research group also employs a high‐performance beam steering metastructure device with ultrathin metasurfaces to aid spatial light modulators.^[^ ^83^ ^]^ This device can significantly enhance the beam steering angle without requiring complex optical configurations. The proposed beam steering superstructure device can enable an intelligent bidirectional optical wireless communication system with a downlink and uplink data transmission rate of 10 Gbps. The signal can be dynamically distributed to nine users and traverse within a field of view angle range of 20° × 20°. This study expands the concept of tunable metasurface devices to intelligent optical wireless communication, offering potential for future 6G applications and paving the way for new metasurface applications. Furthermore, the research team has developed a bidirectional multichannel optical wireless communication system assisted by a polarization‐insensitive beam deflection metasurface, supporting an optical broadcasting system with a capacity of 900 Gbps.^[^ ^84^ ^]^ By leveraging the strong beam deflection capabilities of metasurfaces and the benefits of coherent optical communication, the system's complexity and cost are reduced while providing high transmission capacity. This makes the metasurface beam deflection optical communication system more practical and offers a promising architecture for future high‐performance wireless communication.

### 4.2 Infrared Thermal Imaging

**Figure 8:**
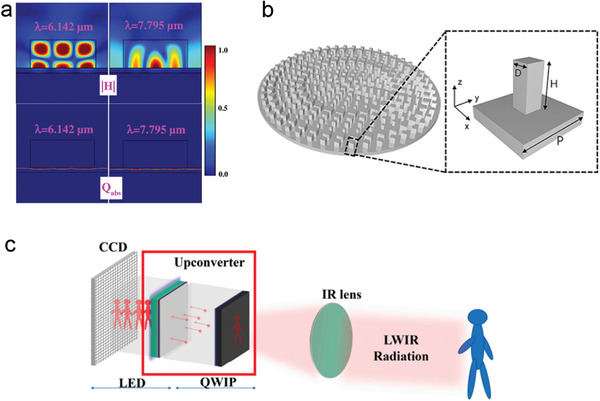
a) *H* and Q_abs_‐field distributions for λ = 6.142 µm and λ = 7.795 µm, respectively.^[^ ^85^ ^]^ Copyright 2020, Chinese Laser Press. b) Schematic diagram of the LFOV metalens.^[^ ^86^ ^]^ Copyright 2023, Optical Society of America. c) Schematic diagram of upconversion pixel‐free imaging.^[^ ^87^ ^]^ Copyright 2023, The Author(s).

Infrared thermal imaging technology plays a crucial role in the military, automotive‐assisted driving, and medical fields. However, its application is limited due to low heat transfer efficiency and slow response time. Chen et al. presented a design and simulation method for a dual‐band perfect absorber based on dielectric metal metasurfaces, gold reflectors, and the thermoelectric material AlN (**Figure** **8**a). They conducted a detailed analysis of the influence of structural parameters on the optical and thermal properties of the absorber.^[^ ^85^ ^]^ The results of the simulation experiment demonstrate the achievement of two narrow perfect absorption peaks, which can be easily adjusted by controlling the structural parameters. Additionally, the system exhibits excellent polarization selectivity and higher thermal conversion and transfer efficiency compared to traditional MIM structures. This work provides valuable guidance and reference for the future design of high‐sensitivity mid‐infrared detectors.

Wang et al. have successfully developed a super optical camera by integrating a large field of view silicon metasurface lens onto an image sensor (Figure 8b).^[^ ^86^ ^]^ The metasurface lens, designed specifically for the long‐wave infrared region, possesses a numerical aperture of 0.81. This lens is capable of focusing light at an incidence angle of up to ±80°, enabling effective wide‐angle thermal imaging of real‐world scenes. This study serves as an important reference for the advancement of large field‐of‐view optical systems and holds enormous potential for applications in areas such as night vision imaging and safety monitoring. Compared to thermal detectors, long‐wave infrared (LWIR) photon detectors have obvious practical advantages due to their high sensitivity and fast response capability. Despite their excellent performance in terms of material and device uniformity, there are issues such as pixel failure caused by differences in the thermal expansion coefficient of materials during chip packaging. Wang et al. initially investigated the optical coupling efficiency (CE) of traditional quantum well‐infrared photodetectors and light‐emitting diodes (QWIP‐LED) devices using the Finite Difference Time Domain (FDTD) method (Figure 8c).^[^ ^87^ ^]^ They then proposed a hybrid metasurface that integrates QWIP‐LED. With the enhanced conversion efficiency, the system is capable of achieving high frame rates above 300 Hz. This work provides solid theoretical guidance for achieving high‐speed up‐conversion pixel‐free imaging.

### 4.3 Infrared Remote Sensing

**Figure 9:**
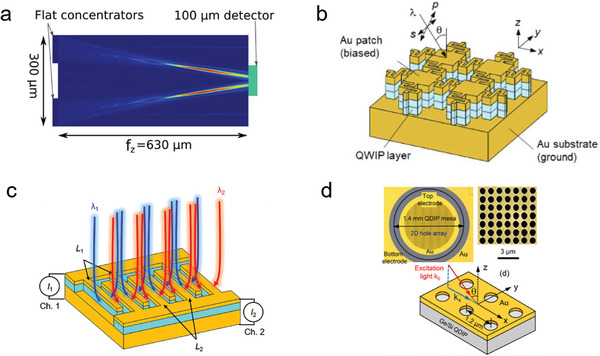
a) Cross section of the simulated electromagnetic field intensity for a lens module from design 1 (the full concentrator consists of eight identical off‐axis metalenses rotated with respect to each other) and design 2 (partial metalenses are used to fill in the empty areas between the modules in design 1) at λ = 4 µm.^[^ ^88^ ^]^ Copyright 2021, The Author(s). b) Schematic diagram of wired etched square detectors.^[^ ^89^ ^]^ Copyright 2021, Optica Publishing Group. c) Schematic diagram of the working principle of a new gas sensor based on dual‐frequency metasurface quantum well‐infrared photodetector.^[^ ^90^ ^]^ Copyright 2020, The Author(s), published by De Gruyter, Berlin/Boston. d) Optical planar images of Ge/Si hybrid photodetectors.^[^ ^91^ ^]^ Copyright 2018, The Author(s).

Infrared detectors have wide applications in capturing medium and long‐wave infrared radiation, playing an irreplaceable role in fields such as military, medical, and environmental testing. However, due to limitations in the physical characteristics of the detectors themselves, there are bottlenecks in terms of their sensitivity, signal‐to‐noise ratio, and operating temperature parameters. In previous studies, scientists have often addressed the issue of signal‐to‐noise ratio improvement in detectors by integrating optical concentrators, allowing for their use at higher temperatures. Wenger et al. reported on an optical concentrator based on a modular design for large‐area metasurfaces (**Figure** **9**a). This design divides each concentrator into multiple sub‐lens modules, with each module being independently designed. This approach effectively enhances design flexibility and reduces computational resource requirements.^[^ ^88^ ^]^ These concentrators are manufactured on GaSb substrates and are highly suitable for integration with backlit infrared detectors, thus improving the signal‐to‐noise ratio and operating temperature of infrared detectors.

Traditional interband transition infrared detectors based on narrow bandgap semiconductors have high sensitivity, but they are difficult to manufacture and often involve highly toxic materials. On the other hand, QWIPs have low toxicity and utilize mature III–V semiconductor group processes, but they typically suffer from weak signals and low optical coupling efficiency. Recently, Japanese scientists have reported a novel multi‐quantum well‐infrared photodetector based on metasurfaces (Figure 9b).^[^ ^89^ ^]^ This groundbreaking design utilizes metal–dielectric–metal (MDM) plasma cavities to achieve detection performance beyond theoretical limits. The study focuses on the impact of the number of quantum wells on the detector's performance. The surface QWIP with three quantum wells (Nw = 3) demonstrates higher detection capability (D*) by effectively reducing noise. It also exhibits excellent performance under different cavity designs, the detection performance under background limited (D*_BG_) can achieve 6.4 × 10^10^ cm Hz^1/2^ W^−1^. This surpasses the theoretical limit (5.3 × 10^10^ cm Hz^1/2^ W^−1^) of interband detectors at a wavelength of 7.0 microns. The findings of this research provide a profound understanding of the working mechanism of metasurface QWIPs, offering valuable insights into the design and manufacturing of high‐performance infrared detectors. Based on this foundational work, the research team further developed a new gas sensor based on dual‐band metasurface QWIPs (Figure 9c).^[^ ^90^ ^]^ This sensor successfully integrates the functions of two detectors and two filters into a single device through resonant photon sorting technology. It effectively overcomes the issues of large system volume, high cost, and limited response speed associated with traditional nondispersive infrared gas sensing technology, which requires two broadband detectors and two independent narrowband filters to capture the target gas absorption line and non‐absorption reference line, respectively. The successful development of this new type of gas sensor has significantly advanced gas sensing technology.

Quantum dot infrared photodetectors (QDIPs) offer significant advantages in suppressing electron–phonon scattering, reducing thermal generation, and minimizing dark current, mainly due to their 3D confinement structure. However, traditional QDIPs have limited absorption ability in the mid‐infrared region, primarily due to the low density of states associated with quantum dots and the limited thickness of the quantum dot absorption layer. This limitation often hinders their practical applications. To address this issue, researchers have explored the use of surface plasma waves (SPWs) to enhance the photocurrent response of QDIPs. Yakimov et al. utilized a 50 nm thick gold film to create periodic circular hole arrays on the film using photolithography, electron beam metal deposition, and stripping techniques as a plasma coupler (Figure 9d).^[^ ^91^ ^]^ Through experimental measurements and computer simulations, it was confirmed that the coupling of mid‐infrared Ge/Si QDIPs with metasurface plasma structures significantly enhances the photoelectric response. Furthermore, a detailed discussion was conducted on the excitation mechanism of mid‐infrared surface plasmon waves. This work presents a novel approach to the design of mid‐infrared photodetectors.

### 4.4 Medical Imaging

Magnetic resonance imaging (MRI) is a powerful medical imaging technique that relies on the precise manipulation of radio frequency (RF) magnetic fields to provide detailed images of the internal structure of the human body. However, the performance of RF coils used in MRI limits their sensitivity and image quality. The application of metasurfaces in MRI is a promising field that can save space inside MR machines and effectively shape the RF field within the scanner. Several studies have demonstrated that using metasurface structures, such as Swiss coil arrays, magnetic induction waveguides, and line dielectrics, to guide electromagnetic signals from receiving coils can effectively enhance the imaging quality of MRI.^[^ ^92^, ^93^, ^94^, ^95^ ^]^

**Figure 10:**
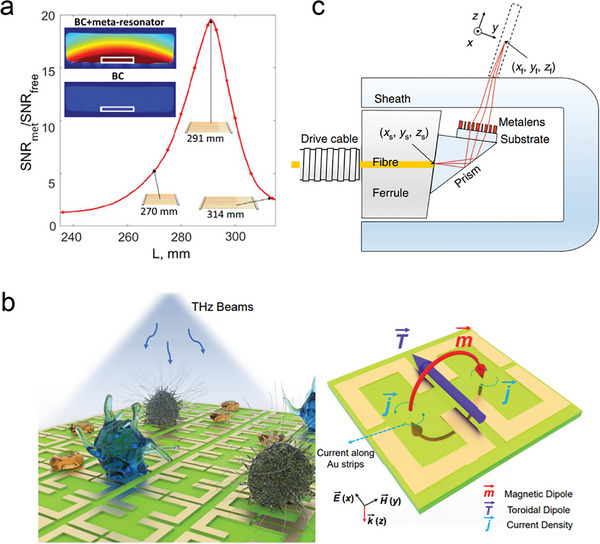
a) The ratio between the SNR with and without a metaresonator measured experimentally is used as a function of L.^[^ ^92^ ^]^ Copyright 2018, John Wiley and Sons. b) Schematic diagram of the metasurface and metaatom.^[^ ^100^ ^]^ Copyright 2021, The Author(s), published by De Gruyter, Berlin/Boston. c) Schematic diagram of nano optical endoscope.^[^ ^101^ ^]^ Copyright 2018, The Author(s).

In 2001, Wiltshire et al. drew inspiration from the natural phenomenon of rainbow butterfly wings and developed a novel “Swiss roll” microstructure magnetic material based on the concept of photonic bandgap materials.^[^ ^96^ ^]^ This material possesses adjustable magnetic permeability within the RF range and exhibits minimal direct current magnetism, thereby preventing interference with the static and audio frequency magnetic fields required for obtaining image and spectral data. This advancement opens up new possibilities for RF flux guidance in MRI. This research paves the way for further development in MRI technology, as evidenced by a series of remarkable studies that followed. Brui et al. developed a novel subwavelength metasurface‐inspired resonator, which can enhance and redistribute the RF magnetic field in MRI scans to improve MRI reception sensitivity and image quality, without occupying additional internal space of the MRI machine (**Figure** **10**a).^[^ ^92^ ^]^ Slobozhanyuk et al. developed an endoscope based on a parallel metal wire array of highly anisotropic metamaterials using metamaterial technology.^[^ ^93^ ^]^ This endoscope is capable of distributing near‐field RF magnetic fields in transmission magnetic resonance imaging. Adjusting the position of the receiving coil can enhance MRI imaging resolution and contrast while reducing scan time. This study demonstrates the potential application of metamaterial endoscopes in MRI, offering a new technological approach to improve imaging quality and reduce scanning time.

These studies demonstrate the potential of metasurface technology in the field of medical imaging, particularly in improving MRI image quality and sensitivity. Furthermore, lung cancer, one of the deadliest diseases worldwide, is currently diagnosed and treated using methods such as optical microscopy, immunohistochemistry, and gene sequencing.^[^ ^97^, ^98^ ^]^ However, these methods are costly and face difficulties in early diagnosis.^[^ ^99^ ^]^ With the introduction of the concept of metasurfaces and the continuous development of metasurface processing technology, low‐cost early diagnosis of lung cancer has become possible. Zhang et al. developed a circular metasurface biosensor based on the terahertz frequency band, which enables rapid, sensitive, label‐free, and cost‐effective detection of lung cancer cells by analyzing their impact on terahertz transmission spectra (Figure 10b).^[^ ^96^ ^]^ This biosensor also facilitates the identification of specific types of lung cancer cells and the formulation of targeted treatment plans. The development of this metasurface biosensor opens up new possibilities for early cancer screening and diagnosis, making it of great significance for the advancement of biomedical science.

Obtaining detailed images of tissue microstructure through endoscopic optical coherence tomography (OCT) is crucial in the diagnosis and treatment of diseases, such as coronary artery and gastrointestinal tract disorders, in modern medicine. Currently, there is a common problem of optical aberration, which makes it difficult to simultaneously achieve both high lateral resolution and depth of focus. Integrated metasurface lenses may serve as an ideal solution to this problem. Pahlevaninezhad et al. incorporated a metasurface lens into OCT to achieve near‐diffraction‐limited imaging (Figure 10c).^[^ ^101^ ^]^ This was accomplished by manipulating the phase of incident light at the sub‐wavelength level and extending the depth range of high‐resolution imaging through specific dispersion designs. This innovative approach enables the simultaneous achievement of excellent resolution and increased imaging depth, thereby offering promising prospects for enhancing OCT applications in disease detection, diagnosis, and related fields. Xiong et al. presented a silicon real‐time hyperspectral imaging chip utilizing reconfigurable metasurfaces, which has potential applications in cerebral hemodynamic imaging.^[^ ^102^ ^]^ The chip features a 155216 (356 × 436) image adaptive microscopy spectrometer, achieving ultrahigh center wavelength accuracy of 0.04 nm and spectral resolution of 0.8 nm. This hyperspectral imaging chip can be seamlessly integrated into almost any commercial camera, enabling a smooth transition between conventional images and spectral images. This research holds significant importance for the advancement and practical implementation of in vivo spectroscopy studies.

### 4.5 Spectrograph

With the continuous advancement of scientific research, the monitoring and comprehensive understanding of various chemical reaction processes has become increasingly important.^[^ ^103^ ^]^ Surface‐enhanced infrared absorption spectroscopy and other spectroscopic techniques are widely employed to monitor the chemical specificity of reaction mechanisms. However, many intermediates associated with chemical reactions have extremely short lifetimes, posing challenges for monitoring. Therefore, the development of efficient spectroscopic techniques holds great importance in achieving a deeper understanding of chemical reaction processes.

**Figure 11:**
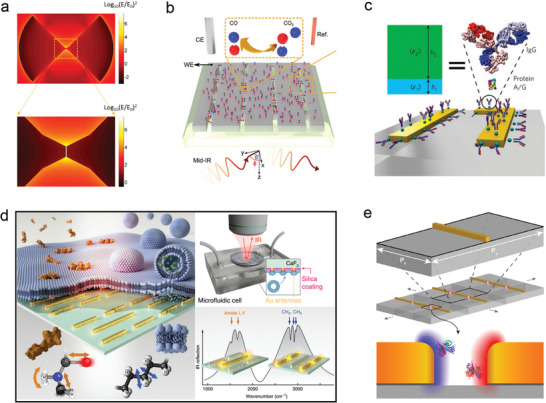
a) Optical properties of surface‐enhanced infrared absorption antennas on a reflective substrate.^[^ ^105^ ^]^ Copyright 2017, American Chemical Society. b) Schematic diagram of Pt‐based nanogroove metasurface for in situ integrated nanophotonic electrochemistry research of catalytic CO oxidation.^[^ ^106^ ^]^ Copyright 2023, John Wiley and Sons. c) Schematic diagram of protein monolayer and bilayer binding to metasurfaces and equivalent dielectric modes.^[^ ^107^ ^]^ Copyright 2011, Springer Nature Limited. d) Schematic diagram of a nanophotonic label‐free biosensor using multi‐resonance mid‐infrared nanoantennas to enhance vibration absorption signals for chemical differentiation of multiple anaerobic cells in biological samples.^[^ ^108^ ^]^ Copyright 2018, Nature Publishing Group. e) 3D models of grating level coupled nanogap antenna units and arrays, as well as schematic diagrams of two types of chain affinity proteins located in nanogap hotspots.^[^ ^109^ ^]^ Copyright 2018, American Chemical Society.

Surface‐enhanced infrared absorption (SEIRA) spectroscopy, as a linear spectroscopic technique, has a cross‐section that detects molecular vibration modes several orders of magnitude larger than surface‐enhanced Raman spectroscopy (SERS).^[^ ^104^ ^]^ However, due to the limitations of electron beam lithography technology, it is difficult to achieve a gap size smaller than 5 nm between metal nanostructures, which hinders the development of SEIRA technology to some extent. Dong et al. developed a novel nano‐gap gold antenna by optimizing the antenna structure (**Figure** **11**a).^[^ ^105^ ^]^ They placed a bow‐shaped gold structure with sub‐3 nm gaps above a reflective substrate. Theoretically, the SEIRA enhancement factor exceeded 10^7^, enabling the detection of very small amounts (≈500–600 molecules) of 4‐nitrothiophenol (4‐NTP) and 4‐methoxythiophenol (4‐MTP) molecules. This contribution provides new ideas for the detection of trace molecules through infrared vibration, improves the limit of single‐molecule SEIRA detection, and offers new possibilities for the application of SEIRA technology in the field of chemical detection. It opens up new paths for the future development of chemical detection and molecular analysis technology. Berger et al. developed a novel hybrid nano photoelectrochemical platform based on platinum nanogroove metasurfaces (Figure 11b).^[^ ^106^ ^]^ This platform enables in situ detection and analysis of molecular signals in electrocatalytic processes through surface‐enhanced infrared absorption spectroscopy measurement. By combining platinum nano groove type metasurfaces with powerful electromagnetic near‐field capabilities, the platform achieves high sensitivity detection of carbon dioxide vibration modes and carbon monoxide weak vibration modes. Compared to traditional platinum films, this metasurface achieves more than a 27‐fold signal enhancement. The developed photoelectrochemical platform holds great potential for future characterization of photoelectrochemical interfaces and in‐depth analysis of chemical reaction processes. Wu et al. introduced a label‐free biosensing technique for structural analysis based on an asymmetric metamaterial plasmon known as Fano resonant asymmetric metamaterials (FRAMMs) (Figure 11c).^[^ ^107^ ^]^ FRAMMs consist of two plasmon antennas along the *y*‐axis and a vertical antenna coupler. This technique enables the measurement of the reflectivity difference (ΔR(ω)) between bare and functionalized substrates. By exploiting the frequency‐dependent infrared response of biomolecules and simultaneously detecting the structure and binding characteristics of biomolecular interactions, it becomes possible to detect and characterize protein monolayers. This provides valuable information on protein thickness and surface binding, thereby contributing to the understanding of conformational dynamics and the prediction of molecular binding processes that sustain life in biological molecules within their natural aquatic environment. Rodrigo et al. have developed a novel label‐free mid‐infrared biosensor utilizing metasurface technology (Figure 11d).^[^ ^108^ ^]^ This biosensor consists of a multi‐resonant mid‐infrared metasurface made up of two sets of gold nano dipoles. By adjusting the length of the dipoles, it becomes possible to effectively distinguish between different biomolecules. When combined with advanced linear regression analysis, this biosensor allows for real‐time monitoring of interactions between lipid membranes, peptides, and other complex molecules. These interactions serve as the foundation for many biological processes, making this research highly significant for both biological research and drug development.

In the aforementioned study, surface‐enhanced infrared absorption spectroscopy (SEIRAS) technology improved the light absorption of near‐surface analytes by utilizing resonant nanoantennas, thereby overcoming the limitations of IR spectroscopy technology in protein detection to a certain extent. Herpin et al. optimized the design of resonant antennas by introducing a novel type of grating‐coupled nanogap (GONG) (Figure 11e).^[^ ^109^ ^]^ By leveraging the strong field enhancement generated by the nanogap and grating cascade enhancement, the detection sensitivity was enhanced to achieve chemically specific detection of proteins, effectively improving the detection limit of SEIRAS technology. This technology allows for the detection of two proteins in each nanogap and the ability to analyze their secondary structure. This study explores the detection limits of protein sensing using a grating‐coupled nanogap antenna design, offering a new approach to enhance the detection sensitivity of SEIRAS.

With the continuous advancement of detection technology and the growing demand for on‐site detection solutions, there is an increasing need for smaller and lighter spectrometers. Yang et al. have developed an ultra‐compact miniature spectrometer utilizing a single‐composition engineered nanowire. This spectrometer is capable of calculating monochromatic and broadband optical reconstruction of the visible light spectrum based on different spectral response functions and measured photocurrents along the length of the nanowire.^[^ ^110^ ^]^ This breakthrough allows for the miniaturization of the entire spectral system to a scale of tens of micrometers, opening up new possibilities for applications in areas such as wearability and implantability. Furthermore, it represents a significant advancement in utilizing other photosensitive nanomaterials for the customized design of ultra‐compact spectral systems.^[^ ^111^, ^112^, ^113^, ^114^, ^115^, ^116^, ^117^, ^118^, ^119^ ^]^

## 5 Outlook and Conclusions

Metamaterials, artificial materials with unique features, have great application potential in the 21st century. The efficient manipulation of electromagnetic waves can be achieved by precisely controlling the microstructural parameters of metamaterials. Unlike natural materials, metamaterials exhibit exceptional capabilities solely from the design of their microstructures, making their design and manufacturing processes highly flexible. Metamaterials have played an important role in emerging technologies such as optical communication, infrared thermal imaging, and medical imaging. Their remarkable ability to manipulate electromagnetic waves highlights their unique advantages and considerable potential for the development of next‐generation high‐performance photodetectors. Consequently, many next‐generation metaphotodetectors have been successfully developed using metamaterials and have been applied in practice.

Next‐generation photodetectors based on metamaterials can effectively achieve wider spectral response ranges, faster light responses, and higher polarization sensitivities, thereby improving their detection performance. In addition, they avoid the need for complex optical systems and offer unique miniaturization advantages that can expand their scope of application. Metaphotodetectors can integrate and realize multiple functions, thereby playing an irreplaceable role in the development of a new generation of multifunctional photodetectors. Metamaterial‐based photodetectors have been successfully applied in various fields, including optical communication, infrared thermal imaging, infrared remote sensing, medical imaging, and spectrometry. This review focuses on the latest progress in the application of metamaterials in photodetectors, discussing their advantages in the field of photodetectors and the practical applications of metaphotodetector devices. It outlines the future development prospects for next‐generation metaphotodetectors, with metamaterials undoubtedly becoming a focal point.

**Figure 12:**
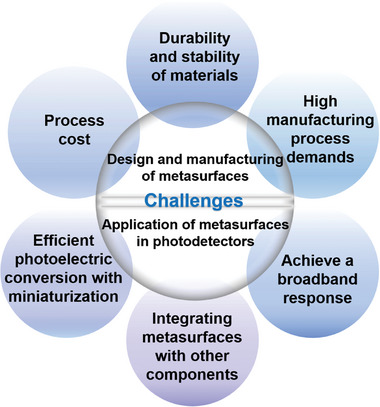
The challenges faced by the next generation of metaphotodetectors.

Although metamaterials have shown considerable promise in the development of metaphotodetectors over the past decade, most of the research discussed in this review is still in its infancy. Consequently, the development of metamaterials and their associated applications still faces considerable challenges (**Figure** **12**).

Because of the intricate microstructural nature of materials, the process involved in the design and manufacturing of metasurfaces relies heavily on high‐precision micro/nano processing technology to accurately produce the desired structural patterns. This results in high manufacturing demands and costs, which pose challenges for large‐scale mass production. Moreover, the choice of materials for manufacturing metasurfaces affects their performance and applications. Their durability and stability must be considered to ensure that their microstructures maintain long‐term operational stability under complex usage conditions. Therefore, in the design and manufacturing process of metasurfaces, multiple aspects such as material selection, process feasibility, durability, and cost must be simultaneously considered.

The application of metasurfaces in photodetector devices requires special materials and structural designs to achieve efficient photoelectric conversion. This process is typically realized through the resonance of electromagnetic waves at a fixed resonance frequency. To achieve a wide frequency response, it is necessary to design multilayer structures or employ collaborative modulation techniques, which may require further optimization of the metasurface structures. Moreover, the integration of metasurfaces with other components or systems can be difficult because of their unique structural characteristics. In addition, the application of metasurfaces in photodetectors often requires a large effective surface area for efficient photoelectric conversion. However, this hinders further miniaturization. Overcoming these contradictions is a major challenge in developing new metaphotodetectors. Nonetheless, we believe that with the continuous efforts of researchers, these problems will be overcome and the large‐scale application of metaphotodetectors will benefit humanity in the near future.

## Conflict of Interest

The authors declare no conflict of interest.
